# The importance of radiation quality for optimisation in radiology

**DOI:** 10.2349/biij.3.2.e38

**Published:** 2007-04-01

**Authors:** CJ Martin

**Affiliations:** Health Physics, Gartnavel Royal Hospital, Glasgow, Scotland

**Keywords:** Radiography, digital radiography, dose calculation, image quality, tube potential

## Abstract

Selection of the appropriate radiation quality is an important aspect of optimisation for every clinical imaging task in radiology, since it affects both image quality and patient dose. Spreadsheet calculations of attenuation and absorption have been applied to basic imaging tasks to provide an assessment of imaging performance for a selection of phosphors used in radiology systems. Contrast, which is an important component of image quality affected by radiation quality, has been assessed in terms of the contrast to noise ratio (CNR) for a variety of X-ray beams. Both CNR and patient dose fall with tube potential, and selection of the best option is a compromise that will provide an adequate level of image quality with as low a radiation dose as practicable. It is important that systems are set up to match the response of the imaging phosphor, as there are significant differences between phosphors. For example, the sensitivity of barium fluorohalides used in computed radiography declines at higher tube potentials, whereas that of gadolinium oxysulphide used in rare earth screens increases. Addition of 0.2 mm copper filters, which can reduce patient entrance surface dose by 50%, may be advantageous for many applications in radiography and fluoroscopy. The disadvantage of adding copper is that tube output levels have to be increased. Application of simple calculations of the type employed here could prove useful for investigating and assessing the implications of potential changes in X-ray beam quality prior to implementation of new techniques.

## INTRODUCTION

The most important choices in optimisation of radiology exposures relate to the amount of radiation to be used and the distribution of photon energies in the X-ray beam or the radiation quality. The latter influences the balance between image quality and dose to the patient because of the manner in which interactions between X-ray photons and tissue vary with photon energy. Radiation quality is determined by the X-ray tube potential selected and the filtration of the X-ray beam. Metal filters are fitted to X-ray tubes to attenuate lower energy photons that are unlikely to reach the image receptor. A filter equivalent to at least 2.5 mm of aluminium is incorporated as standard in medical X-ray tubes and is required by national guidance [1, 2], but additional copper filters may be inserted. Once installed, the filters in radiographic units are seldom altered, but in more complex units used for interventional radiology and cardiology, filters can be added for selected procedures. This may be the choice of the operator or may be determined automatically based on the attenuation of the part of the body being imaged and the procedure programme selected. Tube potential, on the other hand, is selected by the operator for every imaging task either directly or in pre-set programmes. In fluoroscopic procedures, the manner in which the tube potential and current are increased to maintain the dose rate at the image intensifier, is determined by programmes linked to different types of examination.

**Figure 1:**
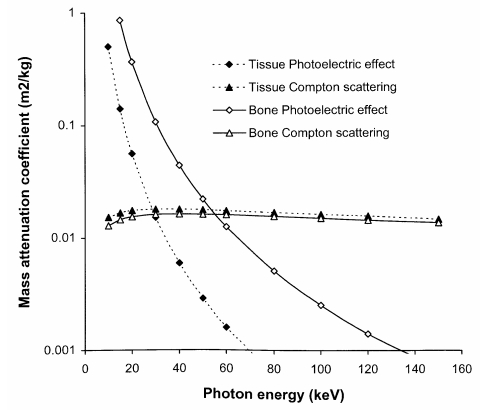
Variation in mass attenuation coefficients for photoelectric absorption and Compton scattering in bone and soft tissue with photon energy [6].

The interaction of X-ray photons with tissue will be reviewed briefly to highlight the implications for X-ray imaging. The processes that are important in the formation of a radiological image are the photoelectric absorption and Compton scattering [3, 4, 5]. The probability of photoelectric interaction increases rapidly with the atomic number of atoms present in the tissue, so it produces good contrast between tissue structures with different elemental compositions. This is demonstrated by the differences in photoelectric mass attenuation coefficients for bone and soft tissue (Figure 1) [6]. Compton scattering is an inelastic process, in which the X-ray photon loses some of its energy and is deflected from its original path, creating a background of random events or noise that degrades the image. The mass attenuation coefficient for Compton scattering is almost independent of tissue composition for diagnostic X-rays, and the probability of interaction depends solely on tissue density. Thus, the contribution of Compton scattering to image contrast is much less than that of the photoelectric effect. For diagnostic X-rays, the number of photons interacting through the photoelectric effect decreases rapidly with photon energy, while the probability of Compton scattering is largely independent of energy (Figure 1). As a result, the proportion of photons interacting via the photoelectric effect changes with the energy spectrum of the X-ray beam, and this affects both image contrast and patient dose [5, 7].

X-ray beams used for medical imaging contain photons with a wide range of energies. The proportion of interactions via the photoelectric effect is higher for X-ray beams containing more low-energy photons (30 keV-50 keV), and so the image contrast is better. However, the greater absorption of energy through the photoelectric effect reduces the proportion of X-rays that is transmitted through the body. As a result, a higher radiation intensity is required and this increases the radiation dose to the patient. More photons in higher energy X-ray beams will penetrate through the body and reach the image receptor. This will tend to give a lower radiation dose, but the image contrast will be poorer. Thus, the choice of photon energy characteristics or radiation quality of an X-ray beam is a crucial component of optimisation in radiology.

Optimisation requires the consideration of both radiation dose and image quality. Radiation dose in this paper is quantified in terms of three quantities; the air kerma incident on the patient, which is proportional to measurements of the dose-area product [8]; the entrance surface dose (ESD), which is the dose to the skin and includes backscattered radiation [8]; and the effective dose [9], which is a quantity computed from simulations that can be estimated from the ESD or dose-area product [8, 10, 11]. Image quality is more difficult to quantify than radiation dose. Detailed imaging performance requires consideration of the ability of the imaging device to reproduce details in terms of the modulation transfer function, and the ability to visualise details against the background noise within the image in terms of the **detective quantum efficiency **or noise equivalent quanta [12, 13]. The most important factor that changes with radiation quality is image contrast and the influence of this on the image can be described in terms of the contrast to noise ratio (CNR). This relates to the contrast or signal difference between larger objects and the image background, but does not incorporate information on resolution. Nevertheless, it is a measure of the aspects in imaging performance, which relate to the choice of X-ray exposure factors. In this study, theoretical simulations have been applied, in order to demonstrate how radiation quality affects both the quality of a radiological image and the radiation dose to the patient. Values for the CNR have been calculated using tissue attenuation properties inserted into a simple attenuation model described and validated in an earlier paper [14]. These have been used to assess how different factors, which alter the X-ray beam quality, influence the imaging performance of radiological imaging systems.

## METHODS

Simple spreadsheet calculations have been performed using data sets for X-ray spectra, filter, phantom and tissue mass attenuation coefficients, and phosphor mass energy absorption coefficients at 1 keV intervals over the range 1 keV to 150 keV [6, 15]. These have been used to predict the responses of radiological imaging systems with different tube potentials and filter options.

The energy absorbed in an image receptor *A(E) *at each photon energy E has been derived from the equation:

(1) $$A(E)=E(1-e^{�-\frac{\mu_{en}(E)}{\rho_{p}}\rho_{p}d_{p}})�$$

where *µ_en_(E)/ρ_ p_, ρ_p_* and *d_p_* are the mass energy absorption co-efficient, density and thickness, respectively, of the image receptor phosphor. Data for the phosphors that have been used in the calculations are listed in Table 1. The caesium iodide phosphor layer can be thicker because the needle-shaped crystals can be aligned so that the needle axes are perpendicular to the image plate, limiting the lateral spread of light that would otherwise degrade the resolution. The thickness of layers of other phosphors need to be limited to about 200 μm in order to maintain resolution.

**Table 1:** Phosphor data used in calculations and radiology application

**Phosphor**	**Density (g cm^-3^)**	**Thickness (μm)**	**Areas of Application**
Gadolinium Oxysulphide	7.34	200	Screen / film systems and indirect digital radiography
Calcium Tungstate	6.062	200	Screen / film systems
Caesium Iodide	4.51	500	Fluoroscopy image intensifiers / indirect digital radiography
Barium Fluoro-Bromide 85% / Iodide 15%	4.8	200	Computed radiography
Selenium	4.25	200	Direct digital radiography

The phosphor sensitivities for X-ray beams of different radiation qualities have been calculated by multiplying equation (1) by the respective number of photons or fluence within each energy interval in the X-ray beam (*ψ(E)*). The results for all photon energies up to the maximum (*Emax*) for each tube potential (*kV*) have been summed to give a measure of the energy absorbed by the image receptor at a particular tube potential ξ*(kV).*

(2) $$\xi (kV)=\sum_{0}^{E\max}{{{}_{}\psi }_{r}}_{}(E)\cdot A(E)$$

where *ψ_r_(E)* is the fluence for photons of energy *E* incident on the image receptor. The quantity ξ*(kV)* has been used as a measure of the image responses of different receptors, such as optical density for film, and light output or signal for digital radiography systems. In order to compare the relative sensitivities *R(kV)* of image phosphors to X-ray beams with different radiation qualities, each result has been divided by the total photon fluence incident on the image receptor.

(3) $$R(kV)=\sum_{0}^{E\max}\psi_{r}(E)\cdot A(E)/\sum_{0}^{E\max}\psi_{r}(E)\cdot$$

Photon fluences have been calculated from data on X-ray spectra generated at different tube potentials and adjusted for attenuation in different filter materials and tissues using tabulated mass attenuation coefficients [15, 6]. The fluence of X-ray photons of energy *E* transmitted through a phantom or patient, and incident on the image receptor *ψ_r_(E)* has been represented by:-

(4) $$\psi_{r}(E)=\psi_{i}(E)\sum_{t}^{T}e^{-\frac{\mu_{t}(E)}{\rho_{t}}\rho_{t}d_{t}}$$

where *ψ_i_(E)* is the X-ray photon fluence incident on the patient, and *µ_t_(E), ρ_t_* and *d_t_* are attenuation co-efficient, density and thickness, respectively, for each layer of tissue or phantom material *t* through which the X-ray beam has passed. The thicknesses of the various tissues within different parts of the chest, abdomen and pelvis traversed by the X-ray beam were measured from sections of adult computed tomography scans, assuming a focus to image receptor distance of 110 cm (Table 2). It was assumed that 80% of the volume taken up by the lung was occupied by air [16].

**Table 2:** Thicknesses of tissue components in sections used in calculations, and of features used to evaluate contrast to noise ratio (mm)

**Examination**	**Chest**	**Chest**	**Chest**	**Chest**	**Abdomen**	**Abdomen**	**Abdomen**
**Part**	**Lung**	**Lung + rib**	**Heart**	**Spine**	**Abdo. 1**	**Abdo. 2**	**Abdo. 3**
Feature	0.8	0.8	3	3		2.5 & 5	
Adipose	27	30	35	22.5	30	60	90
Muscle	62	52	50	100	60	75	90
Soft tissue	-	-	-	12.5	100	105	110
Lung tissue	40	40	19	-	-	-	-
Air	158	158	78	-	-	-	-
Heart muscle	-	-	70	70	-	-	-
Blood	-	-	25	32.5	-	-	-
Bone	-	7	10	37.5	10	10	10
**Total body**	**287**	**287**	**287**	**275**	**200**	**250**	**300**

Values for the air kerma incident on the patient or the image receptor were calculated by substituting *ψ_i_(E)* or *ψ_r_(E)* respectively for *ψ(E)* in the equation:

(5) Kair=∑0Emax⁡ψ(E)⋅E⋅μenρ

where *μ_en_/ρ* is the mass energy absorption coefficient for air. The incident air kerma is used in several examples to show how dose varies with tube potential. Values for patient ESDs for particular examinations were calculated for different X-ray beam spectra by multiplying the incident air kerma by backscatter factors for the appropriate projections [10], and effective doses for a reference patient were derived using tabulated conversion coefficients [8, 11].

The difference in contrast *C(E)* resulting from photons of energy *E* for a feature with linear attenuation coefficient *µ_2_(E)* in an object with attenuation *µ_1_(E)* has been derived from the equation:-

(6) $$C(E)=\frac{I_{1}(E)-I_{2}(E)}{I_{1}(E)}=1-e^{-(\mu_{1}(E)-\mu_{2}(E))d}\approx \Delta \mu (E)d$$

where *I_2_(E)* and *I_1_(E)* are the photon intensities transmitted through the feature and the surrounding area, respectively, and *d* is the thickness of the feature. The factor *Δµ(E).d* in equation (6) was represented by the product of the linear attenuation coefficient for muscle and a small depth of muscle tissue, rather than the differences in tissue attenuation coefficients. Equation (6) has been multiplied by the photon fluence for each energy interval *ψ_r_(E)* and the result summed over the relevant X-ray spectra in order to derive values for the image contrast. The signal can be expressed in terms of the mean number of X-ray photons detected (*N*) by each image pixel, area *a*. Pixel dimensions of 0.14 mm were employed in the calculations.

(7) $$N=\sum_{0}^{E\max}\psi (E)\cdot a$$

The contrast relates to the difference in the mean number of X-ray photons transmitted through the feature (ΔN).

(8) $$\Delta N=\sum_{0}^{E\max}\psi_{r}(E)\cdot a\cdot \Delta \mu (E)\cdot d$$

Since the standard deviation, which describes the fluctuations in quantum noise, is equal to the square root of the number of photons detected (*√N*), the CNR for an ideal image receptor including only quantum noise can be expressed as:-

(9) $$CNR=\frac{\Delta N}{\sqrt{N}}$$

This has been multiplied by the relative sensitivity *R(kV)* (equation 3) to compare imaging performance for different phosphors. This approach assumes that quantum noise makes the dominant contribution to image noise, which will normally be the case for most radiological images. Results were calculated relative to the response at 80 kVp for each spectrum and the tube currents, and so the numbers of photons within the X-ray beams at each tube potential were multiplied by an adjustment factor [*F(kV) = ξ(80) / ξ(kV)*] to equalise the responses of the detectors for each tube potential. For chest radiography in which different tissues are portrayed in the same image, CNRs have been calculated for different regions based on similar levels of air kerma transmitted through the lung fields in order to mimic exposures terminated by an automatic exposure control (AEC) device.

CNR is proportional to *√N*, whereas patient dose is proportional to *N*, so a figure of merit that is independent of the number of photons, and relates solely to differences in the radiation quality, can be defined as the quotient of *CNR^2^*, divided by the system dose.

(10) $$\text{Figure of merit}=\frac{CNR^{2}}{\text{system dose}}$$

Values calculated for the ESD were substituted into equation (10) as the system dose. The simulations reported in this paper relate to the transmitted primary beam and do not take account of scattered radiation reaching the image receptor. Nevertheless, they demonstrate basic relationships between radiation quality, image quality and dose that can be applied in optimisation of radiological systems.

## RESULTS

### Phosphor sensitivity

**Figure 2:**
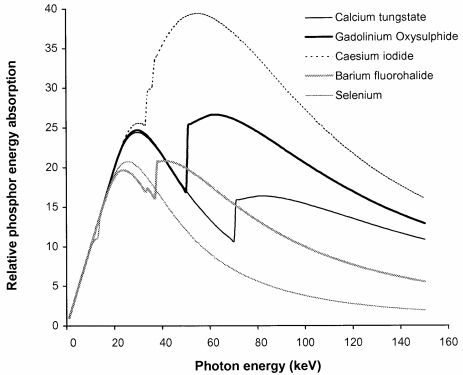
Variation in phosphor sensitivity in terms of absorbed energy A(E) with photon energy for phosphors commonly used in radiology systems, computed using phosphor thicknesses listed in Table 1.

**Figure 3:**
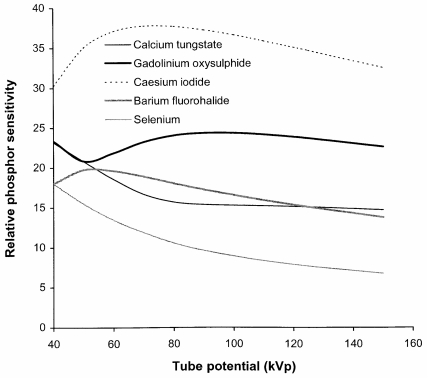
Relative phosphor sensitivity R(kV) to different tube potential X-ray beams for phosphors listed in Table 1. The X-ray spectra applied are those transmitted through 2.5 mm aluminium and 200 mm of water.

The main factors, which affect the radiation quality of an X-ray beam, are the tube potential and the beam filtration. However, there is another factor that influences the quality of the image obtained with different X-ray spectra and that is the variation in the sensitivity of the detector with photon energy. Phosphors or photodiodes are used to convert X-ray energy into light or an electrical signal that can be recorded. Phosphors that are considered in this paper are listed in Table 1, together with the properties used in the calculations and the areas of application. The variations in sensitivities of these phosphors, based on the absorbed energy derived from equation (1), are shown in Figure 2. Relative sensitivities of the same phosphors to X-ray beams corresponding to different tube potentials have been calculated from equation (3) and are portrayed in Figure 3.

Caesium iodide imaging plates are substantially more sensitive than the other systems available, because of the thicker phosphor layers used, so that it should be possible to set these up with image receptor doses of 1.6 μGy to 2.0 μGy, equivalent to a 600 speed index screen/film combination. This is the approach that has been followed in hospitals in the West of Scotland. The sensitivity of gadolinium oxysulphide indirect digital radiography (IDR) systems is similar to that for the screen/film equivalent (400 speed index, 2.5-2.8 μGy detector dose), although the greater dynamic range may be used to achieve some reduction in dose. Direct comparison of the imaging performance for barium fluorohalides, the computed radiography (CR) phosphor, with gadolinium oxysulphide, used in screen/film systems, might suggest that the radiation exposure would need to be 30% to 40% higher to compensate for the lower sensitivity (Figure 3). However, this is offset by the better contrast and dynamic range of digital systems, which should allow satisfactory imaging with a CR system employing a similar dose level to that for a 400 speed index rare earth screen/film system at 80 kVp (2.5-3.0 μGy at the image plate). This approach has been adopted in the West of Scotland with satisfactory results. Selenium, which is a semiconductor photodiode sensitive to X-rays, is employed for direct digital radiography. Selenium performs well at lower photon energies and is used for digital mammography. A higher resolution can be achieved because an intermediate light emitting phosphor is not required.

An important factor that should be taken into consideration is the effect of the difference in the way the sensitivity varies with tube potential. The sensitivity of gadolinium oxysulphide used in rare earth film screen combinations and some IDR systems increases with tube potential by 10% between 60 kVp and 100 kVp, whereas that of barium fluorohalides, which are used in most CR systems, decline by about 17% over this range. This has important implications for the setting up of automatic exposure control (AEC) devices when an X-ray department is converting from screen/film to CR [17]. If an AEC system set up for a rare earth screen/film combination is used for CR systems, it is likely that either images taken with higher tube potentials will have a higher noise level, or exposures at lower tube potentials will be unnecessarily high.

### Tube potential

**Figure 4:**
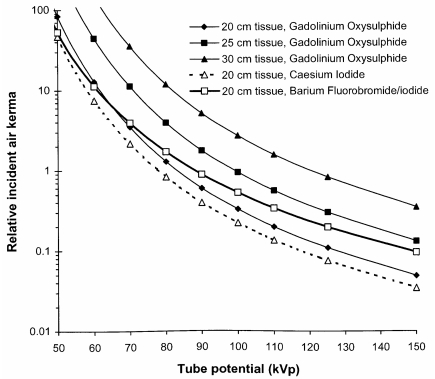
Graphs of incident air kerma against tube potential required to give a similar response for imaging of a 20 cm thick abdominal section of the body with different phosphors, using properties in Table 1. Results are also shown for incident air kerma levels required to obtain images for a gadolinium oxysulphide phosphor system for abdomens with different thicknesses of tissue corresponding to adult patients of varying size (Table 2).

The potential applied to the X-ray tube determines both the maximum photon energy and the proportion of higher energy photons. The optimum potential will depend on the part of the body being imaged, the size of the patient, the type of information required, and the response of the image receptor. Figure 4 shows how the incident air kerma declines with tube potential for imaging conditions adjusted to achieve a similar system response at each tube potential. Results are plotted for several different phosphors for imaging a 20 cm thick abdomen, to show how differences in sensitivity depicted in Figure 3 translate into patient dose. Results are also shown for thicker tissue sections for a rare earth phosphor to demonstrate the increase in dose required for imaging. The change in contrast with the thickness of tissue being imaged for the transmitted beam is small, if the tube potential is kept constant, but the level of scatter from the thicker tissue layers will increase and so the CNR will decline. In addition, the tube potential will normally be increased in order to maintain the dose to the patient at an acceptable level and this will further reduce image contrast. In order to obtain radiographs with a similar ESD for a section of the abdomen that was 250 mm thick, compared to one that was 200 mm thick, the tube potential would need to be increased by about 10 kVp. If the same tube potential was used for both, the ESD to achieve the same image receptor signal would be three times higher for the thicker abdomen. A compromise will normally be chosen, where a slightly higher tube potential is used, accepting some reduction in image contrast, but avoiding the ESD being too high.

**Figure 5:**
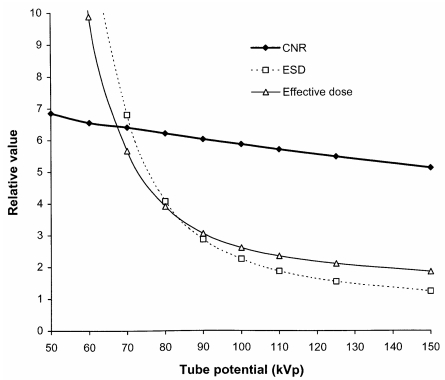
Relative variation in CNR, ESD and effective dose with tube potential for an AP radiograph of the abdomen (Abdo 1, Table 2).

The CNR describes the components of image quality that are affected by the radiation quality and is used here as a measure of imaging performance. The change in CNR to achieve a constant value for the energy absorbed in a phosphor as the tube potential is raised has been calculated using equation (9). Relative changes in the CNR and dose quantities with tube potential are shown in Figure 5. X-ray beams, which contain a greater proportion of photons with energies between 30 keV and 50 keV, give better image contrast (Figure 1), and as a result the CNR gradually declines as the tube potential is raised. However, more of the photons are absorbed in the body, so it is necessary to use a larger radiation intensity in order for sufficient photons to be transmitted through the body to form an image. Relative values for the ESD and effective dose are shown in Figure 5 for an antero-posterior (AP) abdominal radiograph. The effective dose does not fall with tube potential as rapidly as the ESD because lower energy photons make a larger contribution to the absorbed dose at the skin surface than to the doses for tissues deeper within the body.

**Figure 6:**
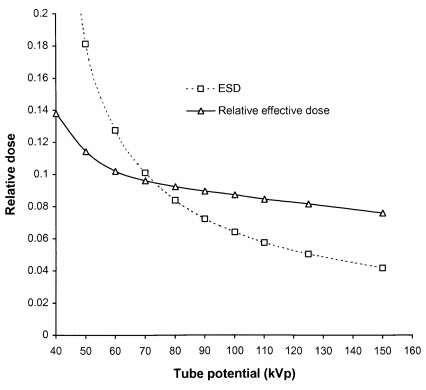
Variation in ESD (mGy) and relative effective dose with tube potential for a PA chest radiograph terminated by AEC chambers behind the lungs (Table 2).

The imaging requirements for chest radiography differ from those for other parts of the body because of the larger difference in attenuation between the lungs and the mediastinum. Chest radiography has been simulated under imaging conditions in which the air kerma incident on the image receptor behind the lung remained constant. This was to represent the termination of the exposure using AEC chambers positioned behind the lungs. Although the air kerma incident on the patient across the whole field is similar, the air kerma incident on the image receptor is much lower in the region of the heart and spine. The dependence of ESD and effective dose on tube potential for a postero-anterior (PA) chest radiograph are shown in Figure 6. Comparatively, the decrease in effective dose with tube potential is lower for PA chest radiographs than for an AP abdomen (Figure 5). This is because the sensitive organs lie closer to the anterior surface of the body, which is adjacent to the image receptor, and the change in dose to deeper tissues with tube potential is lower as the beam has been attenuated by overlying tissues.

**Figure 7:**
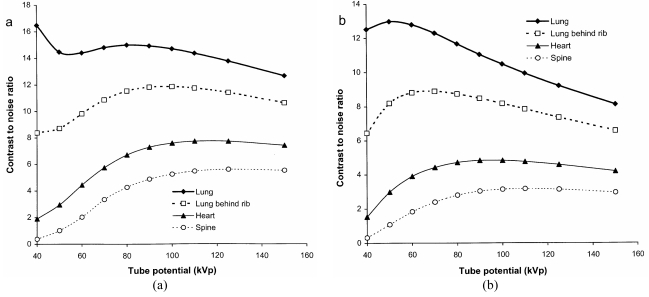
Variation in relative CNR for different parts of a chest image with tube potential for 0.8 mm muscle features in the lung, and 3 mm thick ones in the heart and spine, for an exposure terminated by AEC chambers behind the lungs (Table 2). Results are shown for a) a gadolinium oxysulphide phosphor (rare earth screen) and b) a barium fluoro-bromide/iodide phosphor (CR plate) (Table 1) with an X-ray beam filtered by 2.5 mm of aluminium.

**Figure 8:**
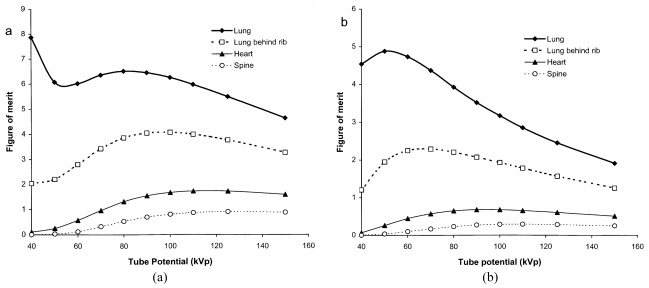
Variation in relative figure of merit with tube potential for different parts of a chest image, for an exposure terminated by AEC chambers behind the lungs. Results are shown for a) a gadolinium oxysulphide phosphor and b) a barium fluoro-bromide/iodide phosphor as in Figure 7.

Values for the CNR have been calculated using different thicknesses of tissue feature in different parts of the image (Table 2) in order to enable the relationships to be viewed on a similar scale. Results are shown for a phosphor used in rare earth screen/film combinations (Figure 7a) and a CR system (Figure 7b). These give an indication of how the visualisation of tissue structure varies in different parts of the chest image and how this changes with tube potential. There are differences in imaging performance with the different phosphors, resulting from the variation in sensitivity portrayed in Figure 3. The CNR for the lung tends to decline with tube potential, but for the gadolinium oxysulphide phosphor, it levels off between 60 kVp and 90 kVp. In practice, the noise is not only due to quantum mottle, but has an anatomic structural component, and for lung tissue, for which the number of photons in the image is higher, the anatomic noise may predominate [18]. Superimposition of a rib degrades the CNR significantly below 80 kVp. The CNR for the heart is higher above 100 kVp, and for the spine tube potentials of 110 kVp to 120 kVp or above give the best CNRs. The performance varies between the different phosphors because the sensitivity of gadolinium oxysulphide increases with photon energy between 60 kVp and 100 kVp, whereas that for barium fluorohalide declines (Figures 2 and 3). Both high and low kV techniques have been used for chest radiography. Higher kV techniques are now generally preferred, as in addition to the CNR in higher density structures, the greater penetration gives a smaller range of beam intensities transmitted through the patient, allowing details to be portrayed in all parts of the image within a narrower exposure range. Figures of merit derived from equation (10) for different parts of a chest image, which take account of image quality and dose, are shown in Figure 8. The conditions in which the figure of merit is higher should represent better imaging performance. Values for the CR phosphor (Figure 8b) are lower, because of the higher dose level required.

### Filtration

**Figure 9:**
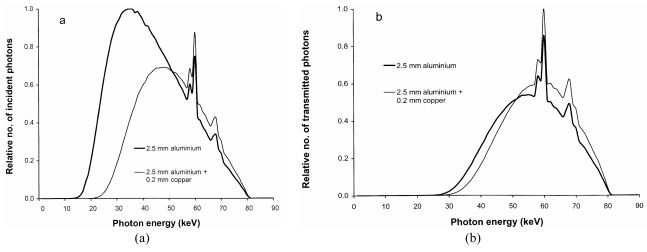
X-ray beams filtered by aluminium and copper, showing the relative proportions of photons a) incident on the patient and b) transmitted through the patient. Data were normalised to give similar energy absorption for a gadolinium oxysulphide phosphor after transmission through 20 cm of tissue (Abdo 1, Table 2).

**Figure 10:**
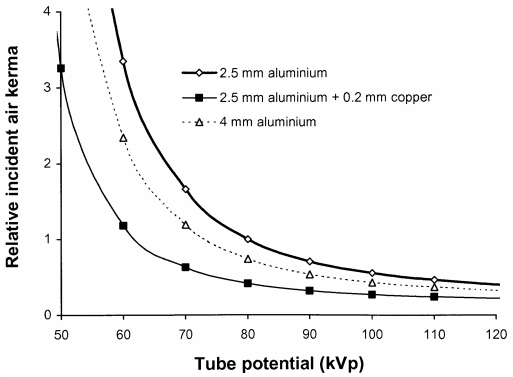
Relative incident air kerma for an abdominal radiograph (Abdo 1, Table 2) against tube potential for X-ray beams using different filter combinations. Results were calculated to give similar energy absorption for a gadolinium oxysulphide phosphor.

Copper filters will absorb a higher proportion, than aluminium, of the photons with energies between 20 and 50 keV, which make a significant contribution to patient ESD (Figure 9). An indication of how the incident air kerma and so the ESD will vary for a radiograph of the abdomen with different aluminium and copper tube filtration options is shown in Figure 10. With tube potentials of 70-80 kV, reductions of over 50% in ESD and 40% in effective dose can be achieved by using a 0.2 mm thick copper filter.

**Figure 11:**
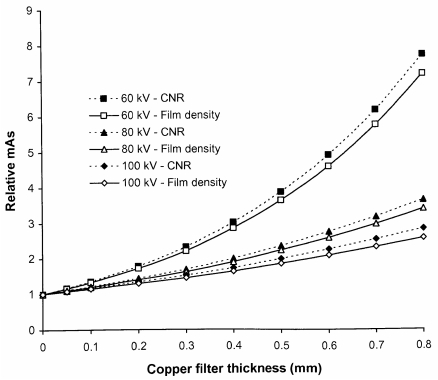
Relative increase in mAs required to obtain a radiograph of an abdomen (Abdo 2, Table 2) with 60 kVp, 80 kVp and 100 kVp X-ray beams, when different thicknesses of copper are added to the X-ray beam, to achieve the same optical density for a gadolinium oxysulphide film screen system (solid lines), and to give the same CNR for a gadolinium oxysulphide digital radiography system (dashed lines).

**Figure 12:**
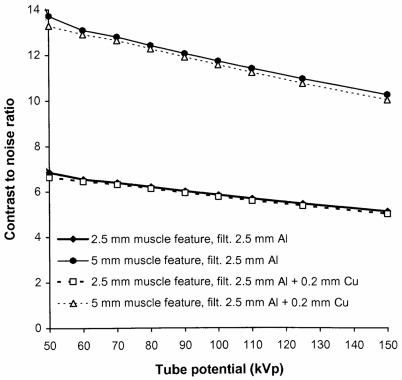
Relative variation of CNR with kVp for 2.5 mm and 5 mm muscle features in a 25 cm thick abdomen (Abdo 2, Table 2), with and without an additional 0.2 mm thick copper filter.

**Figure 13:**
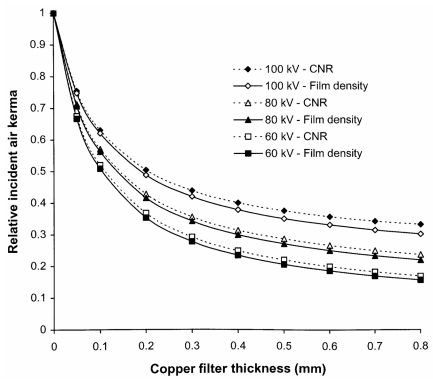
Reductions in incident air kerma against thicknesses of copper filter for the options given in Figure 11, for 60 kVp, 80 kVp and 100 kVp X-ray beams, to achieve the same optical density for a film screen system (solid lines), and to give the same CNR for a digital radiography system (dashed lines).

The disadvantage of using additional filters is that the tube output must be increased in order to compensate for the reduction in photon fluence resulting from attenuation by the extra filters. The tube output would need to be increased by about 50% at 80 kVp to provide the necessary air kerma level to compensate for a filter of 0.2 mm of copper. This may have an impact on the X-ray tube lifetime and also possibly on exposure times. However, copper is much more efficient at removing lower energy photons than the addition of more aluminium. A similar reduction in ESD to that given by the 0.2 mm of copper could only be achieved through the use of 10 mm of aluminium and this would require the tube output to be almost doubled. Thus, copper provides a more effective method for increasing filtration than insertion of more aluminium. The increases in mAs that will be required to achieve the same density level for a rare earth screen/film combination are shown for copper filters of different thickness in Figure 11. The reduction in the proportion of low-energy photons will affect the CNR although the effect is not large and the relative values of CNR for radiographs obtained with and without inclusion of an additional 0.2 mm of copper are shown for abdominal radiographs in Figure 12. For digital radiography, which does not have the limitation in dynamic range imposed by film systems, the mAs could be increased further to achieve a similar CNR. Increases in mAs that would be needed in order to achieve this are also plotted in Figure 11. The corresponding reductions that could be achieved in incident air kerma or dose-area product through inclusion of copper filters of varying thickness are shown in Figure 13. The figure also demonstrates that the most significant reduction is achieved through use of copper filters of 0.2 mm or less. For screen/film radiography, a similar CNR with copper could be obtained by reducing the tube potential by about 5 kVp but this would require a more significant increase in mAs. This approach could be a viable option for paediatric radiography, where exposure factors are lower. Here the use of 55 kVp with an additional 0.2 mm of copper could provide a realistic alternative to a 60 kVp beam.

## DISCUSSION AND CONCLUSION

Radiation quality is of particular importance in the optimisation of radiological imaging in X-ray departments. Variation in sensitivity of the phosphors used in different systems should be considered in determining the radiation intensity required. Barium fluorohalide, the predominant CR phosphor, has a lower energy absorption and so is less sensitive than rare earth screen phosphors for most diagnostic X-ray beams (Figure 3). However, the better contrast and dynamic range of digital systems should allow satisfactory imaging with a CR system employing a similar dose level to that for a 400 speed index rare earth screen/film combination (2.5 μGy-3.0 μGy at the image plate for 80 kVp). The sensitivity of caesium iodide flat plate detectors is 50% greater than that for a rare earth screen/film combination, so that imaging performance equivalent to a 600 speed index system can be attained (1.6 μGy-2.0 μGy detector dose). Sensitivities of gadolinium oxysulphide IDR systems are similar to the screen/film equivalent (400 speed index, 2.5-2.8 μGy detector dose), although the greater dynamic range may be used to achieve some reduction in dose. One aspect of performance that can easily be overlooked during the introduction of digital radiography is the difference in response of the various phosphors with tube potential. It is important that AEC devices are set up to take account of the dependence of the phosphor sensitivity on tube potential at installation, if optimisation is to be achieved. In particular, the sensitivity of barium fluorohalides used in CR systems is significantly lower at higher tube potentials, and this is the reverse of the relationship for rare earth phosphors employed in film cassettes for which sensitivity increases with tube potential (Figure 3).

For all imaging tasks, the selection of tube potential is a compromise to achieve the optimum balance between image quality and dose. High tube potentials are used for thicker parts of the body and adjustments are made for the weight of the patient in order to avoid patient doses being too high (Figure 4). Chest imaging presents a particular challenge. High tube potentials allow all tissues to be imaged within a narrower exposure range, although contrast within the lung is better at lower tube potentials (Figures 7 and 8). The best compromise is probably 100 kVp to 120 kVp, although 90 kVp to 100 kVp may provide a better option for CR, because of the decline in sensitivity at higher tube potentials.

Results of the calculations in this study indicate that incorporation of 0.1 mm or 0.2 mm of copper into most radiological systems will provide advantages in reducing patient dose (Figures 10 and 13), if the X-ray tube is capable of giving the additional output required. The tube current would need to be increased by about 40% to maintain the optical density for film/screen systems (Figure 11). The broader dynamic range of digital radiology systems provides more scope for introduction of dose reduction through use of copper filters, with the facility to increase the exposure to maintain a similar level for the CNR where this is necessary, or further reduce the dose level, where the higher level of image quality is not required. Filter options are now more widely available on new systems and it is important that they are used and their influence understood.

The growth of digital imaging provides new opportunities for optimisation. Calculations of CNR of the type described in this paper for different imaging scenarios can provide the opportunity to evaluate how changes in radiation quality involving filtration and tube potential are likely to affect radiological images. They may assist in investigation and assessment of optimisation strategies prior to their introduction into clinical practice. The greater availability of digital image data should also provide more opportunities for analysis and study of image quality, and so facilitate further optimisation of technique in the future.

## References

[1] Institute of Physics and Engineering in Medicine (2002). Medical and dental guidance notes: A good practice guide to implementing ionising radiation protection legislation in the clinical environment.

[3] Dendy PP, Heaton B (1999). Physics for Diagnostic Radiology. 2 edition.

[4] Hendee WR, Ritenour ER (2002). Medical Imaging Physics. 4 edition.

[5] Martin CJ, Martin CJ, Sutton DG (2002). Interactions of ionising radiations with matter. Practical Radiation Protection in Health Care.

[6] International Commission on Radiation Units and Measurement (1989). Tissue substitutes in radiation dosimetry and measurement.

[7] Martin CJ, Sutton DG, Sharp PF (1999). Balancing patient dose and image quality. Appl Radiat Isot.

[8] Hufton AP, Martin CJ, Sutton DG (2002). Diagnostic Radiology: patient dosimetry. Practical Radiation Protection in Health Care.

[9] International Commission on Radiological Protection (1991). 1990 Recommendations of the International Commission on Radiological Protection. Annals of the ICRP.

[10] Jones DG, Wall BF (1985). Organ doses from medical X-ray examinations calculated using Monte Carlo techniques.

[11] Hart D, Jones DG, Wall BF (1994). Estimation of effective dose in diagnostic radiology from entrance surface dose and dose-area product measurements.

[12] International Commission on Radiation Units and Measurement (1996). Medical imaging - the assessment of image quality.

[13] Martin CJ, Sharp PF, Sutton DG (1999). Measurement of image quality in diagnostic radiology. Appl Radiat Isot.

[14] Doyle P, Martin CJ, Gentle D (2006). Application of contrast-to-noise ratio in optimizing beam quality for digital chest radiography: comparison of experimental measurements and theoretical simulations. Phys Med Biol.

[15] Aitchinger H, Dierker J, Joite-Barfus S (2004). Radiation Exposure and Image Quality in X-ray Diagnostic Radiology.

[16] International Commission on Radiological Protection (1975). Report of the task group on reference man.

[17] Doyle P, Martin CJ (2006). Calibrating automatic exposure control devices for digital radiography. Phys Med Biol.

[18] Samei E, Flynn MJ, Eyler WR (1999). Detection of subtle lung nodules: relative influence of quantum and anatomic noise on chest radiographs. Radiology.

